# Association among dietary magnesium, serum magnesium, and diabetes: a cross-sectional study in middle-aged and older adults

**DOI:** 10.1186/s41043-016-0071-z

**Published:** 2016-10-18

**Authors:** Jie Wei, Chao Zeng, Xiao-xiao Li, Qian-yi Gong, Guang-hua Lei, Tu-bao Yang

**Affiliations:** 1 Department of Epidemiology and Health Statistics, Xiangya School of Public Health, Central South University, Changsha, Hunan Province 410008 China; 2 Health Management Center, Xiangya Hospital, Central South University, Changsha, Hunan Province 410008 China; 3 Department of Orthopaedics, Xiangya Hospital, Central South University, Changsha, Hunan Province 410008 China

**Keywords:** Dietary magnesium, Serum magnesium, Diabetes, Cross-sectional study

## Abstract

**Background:**

A number of studies have reported the association between magnesium (Mg) and diabetes. However, the various conclusions were inconsistent and the data on the Chinese population was limited. The objective of this study was to evaluate the association among dietary Mg, serum Mg, and diabetes in Chinese adults.

**Methods:**

A cross-sectional study that contained 2904 subjects was conducted. Biochemical test results and dietary intakes of subjects were collected for analysis. The adjusted odds ratios (ORs) and the corresponding 95 % confidence intervals (95 % CIs) were used to determine the relationship between Mg status and diabetes by logistic regression.

**Results:**

The prevalence of diabetes of the investigated population was 10.1 %. Dietary Mg intake was not significantly correlated with diabetes (*P* > 0.05). The significant negative association between serum Mg and diabetes existed, and the multivariate adjusted OR was 0.34 (95 % CI 0.24, 0.49) in model 3 for the highest quartile of serum Mg compared with the lowest. The *P* values for trend were all less than 0.001 for the relationship between serum Mg and diabetes. Dietary Mg intake and serum Mg were not significantly correlated in the diabetes population (*P* = 0.936).

**Conclusions:**

Dietary Mg was not significantly correlated with diabetes, while serum Mg was inversely correlated with diabetes in the Chinese population. Meanwhile, dietary Mg intake and serum Mg were not significantly correlated in the diabetes population.

## Background

Magnesium (Mg), as the second most major intracellular cation in the human body, is the basic composition of many enzymes and the cofactor in over 300 enzymatic reactions. Thus, it plays a very essential role in energy metabolism, protein and nucleic acid synthesis, transportation of potassium and calcium ions, signal transduction, and cell proliferation [1–3]. Mg can be found in many foods, especially in whole grains, legumes, green leafy vegetables, and nuts. According to previous researches, Mg intake may be negatively correlated with several chronic diseases, such as metabolic syndrome, diabetes, hypertension, and cardiovascular disease [4–8].

A number of epidemiological and experimental studies have suggested a correlation between Mg and diabetes, but the conclusions were still inconsistent, especially for the relationship between dietary Mg and diabetes. Although quite a few studies [9–16] supported the protective role of higher Mg intake in reducing the risk of diabetes, there were still some studies claiming that the correlation between dietary Mg and diabetes did not exist [17–23]. Meanwhile, the studies denying such a correlation were mainly reported in Asia [19, 21, 23]. In addition, data on Chinese adults are very limited: only two studies reported the relationship between dietary Mg intake and diabetes, and both of them were carried out in East China (Shanghai and Jiangsu) [15, 21]. Several studies have demonstrated the association between serum Mg and diabetes in the Chinese population [24–26]. However, no study specifically focused on the relationship among dietary Mg intake, serum Mg, and diabetes in the Chinese adults.

The objective of this study was to re-evaluate the association between dietary Mg intake and diabetes in the Chinese adults, to assess the average level of Mg intake in the central south area of China, and to test the relationship among dietary Mg intake, serum Mg, and the prevalence of diabetes based on a cross-sectional study with a large sample size.

## Methods

### Subjects

The Xiangya Hospital Health Management Center is one of the largest Health Management Centers in Hunan Province. Participants were recruited while they have their regular health examination. Subjects were selected according to the following inclusion criteria: (1) 40 years old or above; (2) undergoing serum Mg measurement; (3) availability of all basic characteristics, including age, gender, and body mass index (BMI); and (4) completion of a semi-quantitative food frequency questionnaire (SFFQ) about the average consumption of foods and drinks over the past 1 year. This cross-sectional study included 5381 subjects who were undergoing routine checkups including serum Mg measurement from October 2013 to July 2014. Then, 4830 individuals were over 40 years old and available for the basic characteristics. Eventually, 2904 subjects completed the SFFQ and were included in the present study.

The protocol of our study was approved by the Ethics Committees on Research of the Xiangya Hospital, Central South University.

### Dietary assessment

Dietary intake was evaluated by the SFFQ which was particularly designed for the Chinese population. This SFFQ has been validated and used in several previous published studies [27–29]. It contains 63 food items which are popular and commonly consumed in the Hunan Province of China. For each food item, participants were asked how frequently (never, once a month, two to three times a month, one to three times a week, four to five times a week, once a day, twice a day, or more than twice a day) they consumed the food in the past year. The average amount of food intake for each time was asked with six options: less than 100 g, 100–200 g, 201–300 g, 301–400 g, 401–500 g, and more than 500 g. Color pictures of food samples with labeled weights were also given to participants to help them make choices more easily and accurately. The Chinese Food Composition Table [30] was used to calculate the individual composition of macronutrients and micronutrients of the included food items.

### Blood biochemical assessment

All blood samples were drawn after a 12-h overnight fast and were kept at 4 °C until analysis. Serum Mg, blood fasting glucose, high-density lipoprotein cholesterol (HDL), low-density lipoprotein cholesterol (LDL), and triglyceride (TG) were detected on Beckman Coulter AU 5800 (Beckman Coulter Inc., Brea, CA, USA). Subjects with the blood fasting glucose ≥7.0 mmol/L or currently undergoing drug treatment for blood glucose control were regarded as diabetes patients.

### Other covariates assessment

Blood pressure was measured using an electronic sphygmomanometer. Subjects with the systolic blood pressure ≥140 mmHg or diastolic blood pressure ≥90 mmHg or currently using antihypertensive medication were regarded as hypertensive patients. The weight and height of each subject were measured to calculate the BMI. Participants were also asked about their average frequency of physical activity (never, one to two times per week, three to four times per week, five times and above per week) and average duration of physical activity (within half an hour, half an hour to 1 h, 1 to 2 h, more than 2 h). The educational background and smoking and alcohol drinking status were also asked face to face.

### Statistical analysis

Continuous data was expressed as mean ± standard deviation, and category data was expressed in percentage. The Mg intake was classified into four categories based on the quartile distribution: ≤235.28, 235.29–336.06, 336.07–490.76, and ≥490.77 mg/day. Differences in the continuous data were evaluated by the group *t* test (normally distributed data) or the Mann-Whitney *U* test (non-normally distributed data). Differences in the category data were assessed by the *χ* ^2^ test. The odds ratios (ORs) with 95 % confidence intervals (95 % CIs) for the association between Mg intake and diabetes were calculated for each quartile of Mg intake, and the lowest quartile was regarded as the reference category. In order to calculate the adjusted OR for each quartile of Mg intake, three models were adopted for the multivariate logistic analyses: model 1 included age, sex, BMI (≥5, <25 kg/m^2^), and dietary energy intake of participants; model 2 added dietary fiber, calcium, zinc, and iron intake on the basis of model 1; and model 3 added educational level, activity level, hypertension, alcohol and cigarette using condition, and nutritional supplementation on the basis of model 2. The serum Mg was also classified into four categories based on the quartile distribution: ≤0.87, 0.88–0.91, 0.92–0.96, and ≥0.97 mmol/L. Logistic regression was also conducted in three models in order to calculate the adjusted ORs with 95 % CIs for the association between serum Mg and diabetes: model 1 included age, sex, and BMI (≥25, <25 kg/m^2^); model 2 added serum triglycerides, serum LDL, and serum HDL on the basis of model 1; and model 3 added educational level, activity level, hypertension, alcohol and cigarette using condition, and nutritional supplementation on the basis of model 2. Tests for linear trends were conducted using the logistic regression with a median variable of Mg level in each category. The Spearman correlation coefficient was used to detect the relationship between Mg intake and serum Mg for the total population, the diabetes population, and the non-diabetes population, respectively.

All data analyses were performed using SPSS 17.0. A *P* value lower than 0.05 was considered to be statistically significant.

## Results

The basic characteristics of the study population in terms of dietary Mg intake are shown in Table 1. A total of 2904 subjects aged from 40 to 85 years (1650 males and 1254 females) were included in this cross-sectional study. There was no significant difference between included participants and subjects who were excluded due to incompletion of the SFFQ for age, sex ratio, and BMI. The average level of Mg intake was 378.89 mg/day, which was higher than the dietary reference of 350 mg/day recommended by the Chinese society of nutrition [31]. The average level of serum Mg was 0.92 mmol/L. There were 294 subjects of confirmed diabetes, and the overall prevalence of diabetes in this research was 10.1 %. Age, BMI, smoking rate, dietary energy intake, zinc intake, and serum TG were significantly higher in the diabetes population compared to those in the non-diabetes population. Female rate, serum Mg, and serum HDL of the diabetes population were found to be significantly lower than those of the non-diabetes population. Other characteristics were not significantly different between the two comparing groups.

**Table 1:** Characteristics of the study population according to diabetic status

	Diabetic status		*P*
	Diabetes population	Non-diabetes population	
Age	53.92 ± 6.74	52.06 ± 7.26	0.00
Sex (% female)	29.6	44.7	0.00
BMI (kg/m^2^)	26.06 ± 3.06	24.52 ± 3.21	0.00
Educational level (% with or above high school background)	49.0	48.4	0.85
Activity level (h/day)	1.91 ± 3.22	1.90 ± 3.19	0.82
Hypertension (%)	55.8	30.5	0.00
Alcohol using (%)	39.1	37.2	0.52
Smoking (%)	33.3	24.5	0.00
Nutritional supplementation (%)	29.3	33.9	0.11
Energy intake (kcal/day)	1748.72 ± 952.40	1609.35 ± 715.23	0.04
Fiber intake (g/day)	19.81 ± 20.78	18.16 ± 12.66	0.48
Mg intake (mg/day)	410.43 ± 275.10	374.56 ± 197.48	0.05
Ca intake(mg/day)	560.81 ± 551.12	494.42 ± 292.43	0.18
Zn intake (mg/day)	20.96 ± 8.26	19.71 ± 6.40	0.02
Fe intake (mg/day)	33.60 ± 29.27	30.54 ± 16.29	0.07
Serum Mg (mmol/L)	0.89 ± 0.08	0.92 ± 0.07	0.00
Serum TG (mmol/L)	2.79 ± 2.50	1.88 ± 17.71	0.00
Serum LDL (mmol/L)	2.89 ± 1.00	2.95 ± 0.93	0.49
Serum HDL (mmol/L)	1.34 ± 0.34	1.54 ± 0.38	0.00

Data are mean ± standard deviation, unless otherwise indicated. *P* values are for the test of difference between the diabetes population and non-diabetes population

*BMI* body mass index, *Mg* magnesium, *Ca* calcium, *Zn* zinc, *Fe* iron, *TG* triglyceride, *HDL* high-density lipoprotein cholesterol, *LDL* low-density lipoprotein cholesterol

The results of the multivariable-adjusted relationship between dietary Mg intake and diabetes are listed in Table 2. After the adjustment by age, sex, BMI, dietary energy intake, fiber intake, calcium intake, zinc intake, iron intake, educational level, activity level, hypertension, alcohol drinking and smoking status, and nutritional supplementation, the ORs and their 95 % CIs did not show a significant correlation between dietary Mg intake and the prevalence of diabetes. The *P* values for trend of the three models also indicated an insignificant association between dietary Mg intake and diabetes (*P* > 0.05).

**Table 2:** Multivariable-adjusted relationship between magnesium intake and diabetes

	Quartiles of Mg intake				*P* for trend
	Q1 (lowest)	Q2	Q3	Q4 (highest)	
Median Mg intake (mg/day)	179.95	284.46	399.42	612.95	–
No. of subjects	726	726	726	726	–
No. of cases	68	69	62	95	–
^a^Model 1	1.00 (reference)	0.85 (0.59, 1.23)	0.71 (0.48, 1.05)	1.00 (0.66, 1.51)	0.82
Model 2	1.00 (reference)	0.86 (0.59, 1.25)	0.74 (0.49, 1.13)	1.18 (0.67, 2.06)	0.54
Model 3	1.00 (reference)	0.86 (0.59, 1.26)	0.77 (0.50, 1.19)	1.18 (0.66, 2.13)	0.54

Data are adjusted OR (95 % CI), unless otherwise indicated

^a^Model 1 included age, sex, BMI (≥25, <25 kg/m^2^), and dietary energy intake of participants; model 2 added dietary fiber, calcium, zinc, and iron intake on the basis of model 1; model 3 added educational level, activity level, hypertension, alcohol and cigarette using condition, and nutritional supplementation on the basis of model 2

The concentration of serum Mg was 0.89 ± 0.08 mmol/L in the diabetes population, which was significantly lower than that in the non-diabetes population (0.92 ± 0.07 mmol/L, *P* < 0.001). The results of the multivariable-adjusted association between serum Mg and diabetes are displayed in Table 3. According to the results of the multivariable logistic regression, there was a strong inverse correlation between serum Mg and diabetes. The multivariate adjusted ORs for diabetes were 0.38 (95 % CI 0.27, 0.54) in model 1, 0.34 (95 % CI 0.24, 0.50) in model 2, and 0.34 (95 % CI 0.24, 0.49) in model 3 for the highest quartile of serum Mg compared with the lowest. The *P* values for trend were less than 0.001 in all the three models.

**Table 3:** Multivariable-adjusted relationship between serum magnesium and diabetes

	Quartiles of serum Mg				*P* for trend
	Q1 (lowest)	Q2	Q3	Q4 (highest)	
Median Mg concentration (mmol/L)	0.84	0.90	0.94	1.00	–
No. of subjects	827	672	710	695	–
No. of cases	130	57	58	49	–
^a^Model 1	1.00 (reference)	0.48 (0.34, 0.67)	0.45 (0.32, 0.62)	0.38 (0.27, 0.54)	0.00
Model 2	1.00 (reference)	0.48 (0.34, 0.68)	0.45 (0.32, 0.63)	0.34 (0.24, 0.50)	0.00
Model 3	1.00 (reference)	0.48 (0.34, 0.68)	0.45 (0.32, 0.63)	0.34 (0.24, 0.49)	0.00

Data are adjusted OR (95 % CI), unless otherwise indicated

^a^Model 1 included age, sex, and BMI (≥25, <25 kg/m^2^); model 2 added serum triglycerides, serum LDL, and serum HDL on the basis of model 1; model 3 added educational level, activity level, hypertension, alcohol and cigarette using condition, and nutritional supplementation on the basis of model 2

The relationship between Mg intake and serum Mg was also tested by the Spearman correlation coefficient. The association between Mg intake and serum Mg was positively correlated in the total study population (*r* = 0.049, *P* = 0.009). After stratification by diabetes, a significantly positive correlation was only found in the non-diabetes population (*r* = 0.060, *P* = 0.006), but not in the diabetes population (*P* = 0.936).

## Discussion

A cross-sectional study with a large sample size (2904 subjects) was conducted in the Hunan Province of China, aiming at exploring the relationship among dietary Mg intake, serum Mg, and diabetes in Chinese adults. According to our knowledge, this is the first report about the association among the three elements in the Chinese population. The prevalence of diabetes of the study population was 10.1 %, which was very close to the estimated level (11.6 %) investigated on a representative sample of Chinese adults in 2010 [32]. This could suggest a good representativeness of this study population. In this research, it was found that dietary Mg intake was not significantly correlated with diabetes, but serum Mg was in a strongly inverse correlation with diabetes. Meanwhile, there was not a significant association between dietary Mg intake and serum Mg in the diabetes population.

Most studies have suggested a significantly inverse correlation between dietary Mg and diabetes [9–16], but there were also some researchers concluding that the relationship between dietary Mg and diabetes was insignificant [17–23]. Such inconsistency was mainly found in Asia. Three studies [19, 21, 23], together with this research, indicated that dietary Mg was not significantly correlated with diabetes in Asian populations. Dietary Mg is closely associated with other healthy lifestyles and dietary factors, such as fiber and calcium intake [33], and therefore, it seems inappropriate to explain the effect of Mg intake based on its association with diabetes alone. Previous studies have built various multivariable models to predict the effects of Mg intake on the risk of diabetes; however, most outcomes were not adjusted by fiber or calcium intake [9–16]. Nanri et al. [19] suggested that the lean body composition of the Asian population might be one of the primary causes of the relatively less significant association between Mg and diabetes. Since Mg may affect insulin resistance, the Asian population probably has less insulin resistance compared to the Western population due to the leaner body composition. Meanwhile, the differences between this study and other studies targeting non-Asian populations may be attributed to the general dietary habits. In the Chinese diet, the major source of Mg is cereals, such as white rice; this is very different from Western dietary habits. The study population of this research might have a higher Mg intake from cereal products compared to the Western populations, which resulted in the inconsistent association between dietary Mg and diabetes.

The strong inverse association between serum Mg and diabetes observed in this study is consistent with the findings of several other epidemiological studies [17, 20, 26, 34–36]. Previous studies have proved that a low level of Mg would damage insulin receptor function and decrease insulin sensitivity [37]. Serum Mg is always an important pointer of Mg deficiency, though extracellular Mg only accounts for 1 % of the total body Mg [38]. On the opposite, diabetes could result in hypomagnesemia. The case-control study conducted by Lecube et al. [35] suggested that diabetes was the main factor accounting for the low concentration of serum Mg observed in morbidly obese subjects, because insulin deficiency or resistance could inhibit Mg re-absorption and accelerate its excretion in the kidney [1, 39]. The urinary Mg was not measured in the present study. However, it was found that Mg intake was not significantly associated with diabetes. Since Mg homeostasis is mainly regulated by the balance between intestinal absorption and renal excretion [38, 39], a possible explanation for the low concentration of serum Mg in diabetes subjects could be the increased renal Mg excretion which was caused by insulin deficiency or resistance.

The present study also examined the association between dietary Mg and serum Mg. We also found that the correlation between dietary Mg and serum Mg did not exist in diabetes subjects. Although there were significantly positive associations between dietary Mg and serum Mg in the total population and non-diabetes population, the correlation coefficients were extremely low (*r* = 0.049 and *r* = 0.060, respectively). We could not be sure whether this statistical significance resulted from the large sample size or the actual association between dietary Mg and serum Mg.

As a major strength, this is the first study that evaluated the relationship among dietary Mg, serum Mg, and diabetes with a large sample size of the Chinese population. According to the results, dietary Mg was not significantly associated with diabetes, but serum Mg was in a significantly inverse correlation with diabetes. In addition, the relationship between dietary Mg and serum Mg in the Chinese population was also tested for the first time, and it revealed that the association between dietary Mg and serum Mg was not significant in diabetes subjects. The effects of increased insulin resistance on Mg homeostasis in diabetic subjects might be a possible explanation for these findings.

There were also several limitations in the present research. Firstly, this cross-sectional study was unable to explain the causal relationship among dietary Mg, serum Mg, and diabetes, but based on the results, it could be speculated that Mg and diabetes might interact with each other. This hypothesis needs to be confirmed by further prospective studies. Secondly, the effects of Mg supplements on serum Mg or diabetes were not evaluated. According to previous studies [40, 41], Mg supplements may affect the serum Mg and/or glucose metabolism. Thirdly, the urinary Mg level was not measured either, further studies should combine the factor of urinary Mg to confirm the hypothesis that insulin resistance was responsible for the low concentration of serum Mg in diabetes subjects.

## Conclusions

In conclusion, the main finding of this study was that dietary Mg was not significantly correlated with diabetes, while serum Mg was in a strongly inverse correlation with diabetes in the Chinese population. Meanwhile, dietary Mg and serum Mg were not significantly correlated in the diabetes population. According to our findings, it could be speculated that the low concentration of serum Mg in diabetes subjects was mainly caused by the enhanced insulin resistance, which could increase Mg excretion in urine.
